# Chemometric Discrimination of *Cichorium glandulosum* Boiss. et Huet and *Cichorium intybus* L. via Their Metabolic Profiling, Antioxidative, and Hypoglycemic Activities

**DOI:** 10.3390/foods12040901

**Published:** 2023-02-20

**Authors:** Maoru Li, Guoyong Xie, Yadong Ding, Ji Ma, Qiuyan Liu, Yuqin Wang, Zan Peng, Jianbo Sun, Jing Shang

**Affiliations:** 1 Jiangsu Key Laboratory of TCM Evaluation and Translation Research, School of Traditional Chinese Pharmacy, China Pharmaceutical University, Nanjing 211198, China; 2 Department of Resources Science of Traditional Chinese Medicines, School of Traditional Chinese Pharmacy, China Pharmaceutical University, Nanjing 211198, China; 3 Department of Natural Medicinal Chemistry, China Pharmaceutical University, Nanjing 211198, China

**Keywords:** *Cichorium glandulosum* Boiss. et Huet, *Cichorium intybus* L., HPLC-QTOF-MS, plant metabolomics, activity comparison

## Abstract

*Cichorium glandulosum* Boiss. et Huet (CG) and *Cichorium intybus* L. (CI) are widely used as the main raw material of functional food with hepatoprotective and hypoglycemic effects. Due to the lack of comparison on the chemical ingredients and efficacy, they were often used imprecisely and interchangeably. It is necessary to distinguish between them. With the plant metabolomics based on high-performance liquid chromatography coupled with quadrupole time-of-flight mass spectrometry (HPLC-QTOF-MS) and multivariate chemometric techniques, the chemical ingredients were characterized and 59 compounds between CG and CI were classified. As for antioxidative and hypoglycemic activities in vitro, CI extraction exhibited better antioxidant activity than CG, while CG extraction showed stronger hypoglycemic activity. Furthermore, a bivariate correlation between the chemical composition and efficacy of the extract was also analyzed, and three differentially strong correlation components between CI and CG were prepared, and the antioxidative and hypoglycemic efficacies were compared in vivo and different active phenotypes were obtained. Finally, we revealed chemical and biological differences between CG and CI, providing a basis for achieving better quality control and developing more effective functional foods.

## 1. Introduction

*Cichorium glandulosum* Boiss. et Huet (CG) and *Cichorium intybus* L. (CI) are perennial plants from the Cichorium genus, Asteraceae family. In China, these plant species were admitted to the Chinese Pharmacopoeia. The dry aerial part and root are collectively referred to as chicory and used for clearing liver and bile, stomach strengthening, digestion, and diuretic swelling [1]. They have also been wildly used as fodder for livestock and poultry, vegetables, spices, and even as herbal medicine in recent years [2]. The extractions of CG and CI have been reported to exert hypoglycemic, lipid-lowering, antimicrobial and hepatoprotective effects [3,4]. They are, simultaneously, functional foods with various active ingredients originating from CG or CI and have become very popular in the food industry, mainly carrying out the hepatoprotective and hypoglycemic effects. CG and CI are valuable sources of bioactive substances in new food products, especially since inulin, sesquiterpene lactones, and phenolic compounds [5,6] are found in the plant roots, leaves, seeds [7] and flowers. This research suggests that CG and CI may have potential health effects, which makes them potentially valuable resources for food ingredients, functional foods, beverages, and dietary supplements. For example, health products lsuch as Biodrain and capsules of Astragalus Chicory and Fructus Lycii are now widely used.

Based on the wide range of applications, further research has been done on these. Although they are uniformly listed as chicory in the Chinese pharmacopoeia, the two species are similar in many ways but still possess some distinguishing features. Geographically speaking, CI is widely distributed in Europe, Western Asia, Australia, North America, and Regions of China [8], while CG is mainly distributed in Aksu, Kashgar Prefecture, and other plains and oases in Xinjiang, China. Regarding morphology, for instance, CG stems and leaves are covered with villi, while CI has a smooth appearance. Even so, CG and CI are often confusing to use in the market, which affects the ingredients and health-care efficacy of their products.

Modern studies have shown that CG extraction can regulate lipoidemia [9] and glycemia [10], which is mainly owing to its flavonoids and phenylpropanoids, such as caftaric acid and kaempferol-3-O-glucuronoside. CI extraction has hepatoprotective [11] and antioxidative effects [12] mainly associated with 11β,13-dihydrolactucin and lactucopicrin. Because of the confusion between the two species, the origins of species in these studies may be inaccurate, with serious implications for their future product development. In our literature review, we found that few studies have been able to simply compare the content of certain chemicals between CG and CI. In order to achieve better quality control and develop more effective functional foods, it is necessary to systematically compare and evaluate the composition and efficacy of CG and CI.

In this study, a standard chromatographic fingerprint with a simple and reliable analytical method of CG and CI by HPLC was developed. Metabolites in CG and CI were qualitatively and quantitatively identified by untargeted metabolomics. Additionally, we investigated how chemical differences between CG and CI lead to distinct antioxidative and hypoglycemic activities. The results not only provide chemical information for product quality evaluation but also provide a strong basis for future food product development.

## 2. Materials and Methods

### 2.1. Chemicals

Methanol, acetonitrile, and acetic acid were purchased from Merck (Darmstadt, Hesse, Germany) and TEDIA Company Inc. (Fairfield, OH, USA). High-purity water was acquired from Hangzhou Wahaha Group (Hangzhou, China). The remaining chemical reagents were purchased from Sinopharm Co. Ltd. (Shanghai, China). P-nitrophenyl-α-D glucopyranoside (pNPG), 1,1-diphenyl-2-picryl-hydrazyl (DPPH), 2,20-azino-bis-3-ethylbenzthiazoline-6-sulphonic acid (ABTS+), acarbose, glucose (GLU), alloxan (ALX), and L(+)-ascorbic acid (Vc) were purchased from Shanghai Yuanye Bio-Technology Co., Ltd., (Shanghai, China).

### 2.2. Sample and Sample Preparation

Ten batches of CG and ten batches of CI samples were collected from XinJiang, China for analysis, and the source of informationias listed in the Table S1 (Supporting Materials). Professor Minjian Qin (China Pharmaceutical University) identified the herb species. Voucher samples deposited at the Department of Pharmacognosy, China Pharmaceutical University, Nanjing, China. 

CG and CI were pulverized with a pulverizer, and sample was accurately weighed (1 g) and placed in a round-bottomed flask. After 20 mL of methanol water (7:3), the mixture was extracted by ultrasound (500 W) for 50 min, then added methanol water (7:3) as required, adjusting to the initial weight. Extractions were then filtered through a microfiltration membrane (0.22 μm) to obtain the filtrate. The ample solution was stored in the sample injection bottle for HPLC and LC-MS analysis. Quality control (QC) samples were prepared by mixing equal volumes of each sample analyzed in this study. 

The powder was decocted in 70%methanol (1:20, 1.5 h). After concentration and centrifugation, extraction supernatant, filtered with microfiltration membrane (0.45 μm), and then further enriched by preparative liquid chromatography.

### 2.3. HPLC Apparatus and Chromatographic Conditions

The HPLC fingerprint analysis was run on an Agilent 1260 HPLC series (Agilent Technology, Santa Clara, CA, USA), equipped with a low pressure mix binary pump, an auto sampler, a diode array detector (DAD), and an online degasser. Chromatographic separation was carried out at 35 °C on the Agilent ZORBAX SB-C18 column (4.6 mm × 250 mm, 5 μm). The mobile phase was composed of 1% acetic acid aqueous solution (A) and methanol (B) in a gradient elution mode (0–5 min, 10–15% B; 5–15 min, 15–30% B; 15–30 min, 30–45% B; 30–50 min, 45–45% B) at a flow rate of 1.0 mL·min^−1^. The UV absorbance was monitored at 258 nm. It was kept constant during all the batches. 

### 2.4. LC-QTOF MS Apparatus and Chromatographic Conditions

For the identification of chemical components from samples, an analysis was performed using an Agilent series 1290 Infinity HPLC instrument coupled with an Agilent 6530 Q-TOF mass spectrometer (Agilent Technologies, Santa Clara, CA, USA). Gradient elution procedure is consistent with HPLC. The flow rate was 0.5 mL/min, and the injection volume was 5 μL. Mass spectrometric detection was performed in negative electrospray ionization (ESI) mode. The operating parameters were as follows: capillary voltage, 4000 V; dry gas (N_2_) flow rate, 8 L/min and the temperature, 325 °C; sheath gas flow rate, 12 L/min and the temperature, 350 °C; nebulizer, 40 psi. Mass spectra were recorded over the mass range of *m/z* 50–1700 with accurate mass measurement of all mass peaks. The auto MS/MS mode was set to obtain abundant structural information. All the data acquisition were controlled by Agilent Mass Hunter software (version B.01.03 Build 1.3.157.0 2, Agilent, USA). QC and blank samples were analyzed in the batch, and they were placed every 5 randomized sample injections.

### 2.5. Data Pretreatment and Multivariate Data Analysis

The chemical similarity of chromatographic fingerprints was calculated with the “Similarity Evaluation System for Chromatographic Fingerprint of Traditional Chinese Medicine (2010 A Version, Committee of Chinese Pharmacopeia, China)” software [13]. It was used to assess the quality consistency of the CG and CI. Similarity parameter quantified the difference between two fingerprints. The LC-MS raw data were converted to common data format (mzML) files for the untargeted metabolic analysis using a conversion software program MS converter [14]. After peak alignment, peak matching and peak extraction, a three-dimensional matrix consisting of peak intensity information, variables characterized by retention time (t_R_) and *m/z* values was formed. The variables presented in at least 80% of either group were extracted [15]. Metabolite ions with RSD% less than 30% in QC samples were normalized for further data processing. Then he principal component analysis (PCA), and orthogonal partial least squares discriminant analysis (OPLS-DA) were carried out to further identify the data patterns. The PCA was conducted to detect the samples intrinsic variation. The OPLS-DA was used to distinguish the differences in metabolic profiles between sample groups. The characteristic chemical markers were selected by VIP > 1 and *p* < 0.05 in the moderated t-test. Structure annotation of the potential chemical markers was achieved by the characteristic fragments and the fragmentation patterns confirmed by MS/MS. Compounds were tentatively identified using a combination of accurate mass and MS/MS fragmentation patterns and were compared against published literature on their chemical composition publicly available in online metabolite databases Metlin [16], MassBank, and HMDB [17]. A heat map was used to visualize the variation in the levels of the potential chemical markers in all of the CG and CI extractions.

### 2.6. In vitro Activity Assay

#### 2.6.1. Assays for Antioxidant Activities In Vitro

In vitro antioxidant activity was assessed by 1,1-diphenyl-2-picrylhydrazyl (DPPH) [18] radical scavenging assay and ABTS radical cation (ABTS+) [19]. In DPPH assay, 2 mL of various dilutions of the samples were mixed with 1mL of a 0.25 mM methanol solution of DPPH. The mixtures were kept at room temperature in the dark for 30 min, and absorbance was measured at 517 nm using the UV spectrophotometer to reflect the scavenging capacity. The ABTS+ was prepared by reacting 7 mM stock solution of ABTS with 2.45 mM potassium persulfate and allowing the mixture to stand in the dark at room temperature for 12 h before use. The ABTS+ solution was diluted with methanol to an absorbance of 0.800 ± 0.05 at 734 nm. Next, 2.85 mL of the methanol solution was added to 0.15 mL of different concentrations of the samples and the absorbance was recorded at 734 nm after mixing up to 10 min. L(+)-ascorbic acid (Vc) was used as the positive control. The radical scavenging activity of the tested samples expressed with IC_50_ value.

#### 2.6.2. Assays for Hypoglycemic Activities In Vitro

Assays for α-glucosidase inhibition properties (PTG) using the method described by Lei Wang et al. [20]. CG and CI solution with different concentrations were prepared using a phosphate buffer (100 mM, pH 6.9), and 100 μL of α-glucosidase (0.5 U/mL) was added to 100 μL of sample solution and incubated at 37 °C for 10 min. Then, 100 μL of pPNG (5 mM) was added to each reaction vial and further incubated at 37 °C for 20 min, and 1 mL sodium carbonate (1 M) was added and the reaction was terminated. The absorbance value was then measured at 405 nm. The pharmacological inhibitor, acarbose, was introduced as a positive control. The hypoglycemic activities of the test samples are expressed in IC_50_ values.

### 2.7. In Vivo Activity Assay

#### 2.7.1. Antioxidant Activity in Larval Zebrafish

The oxidation-sensitive fluorescent probe DCFH-DA was used to detect intracellular ROS generation in zebrafish larvae [21]. The wild-type AB-line larvae zebrafish were developed to 15 dpf. On the 15th day, the larval zebrafish were used for further treatment. Kaempferol-3-O-glucuronoside (CGA), 15-deoxylactucin-8-sulfate, and 8-deacetylmatricarin-8-O-sulfate (CIA) were dissolved directly into embryo water at a concentration of 5, 10 and 20 ug/mL and larvae were pretreated for 20 h. Then, larvae were induced the oxidant stress by H_2_O_2_ (2 mM) for 4 h. DCFH-DA can as an indicator of ROS, and a 10 mM solution of DCFH-DA was prepared by dissolving it in DMSO. Larval zebrafish were incubated with 10 μM DCFH-DA (diluted with embryo water) for 30 min at room temperature in the dark. The larvae were washed with embryo water 3 times and kept in CMC-Na (4%) on a glass slide for fixation. The image was captured immediately using a fluorescence stereoscope (Olympus SZX16). ImageJ software (https://imagej.net) was used to measure whole-body fluorescence [22].

#### 2.7.2. Hypoglycemic Effect Determination on Zebrafish Diabetic Model

Zebrafish at 5 dpf were randomly divided and transferred into 6-well plates. The density was 30 fish per well. Zebrafish were cultured in water with 10 mg/mL GLU and 0.025 µM ALX for 24 h to obtain diabetic model [23]. Experimental groups were treated with different concentrations of CGA and CIA (5, 10, and 20 µg/mL), 10 mg/mL GLU were administered at the same time. All groups were incubated in an incubator at 28 ℃ for 24 h, and then zebrafish were collected in 1.5 mL centrifuge tubes. Each tube contained 10 fish, with 3 tubes per group. Determination of glucose content according to the glucose kit protocol was carried out.

All the animal experiments were approved by Ethical Committee of China Pharmaceutical University (SYXK(SU)2021-0010) and Laboratory Animal Management Committee of Jiangsu Province. All the experiments followed the Jiangsu Provincial standard ethical guidelines in using experimental animals under the ethical committees mentioned above.

### 2.8. Statistical Analysis

All results were expressed as the means ± standard deviations (SDs) of at least three independent measurements. Graph Pad PRISM [24] (Graph Pad Software, United States, USA) was used for comparing the treatment group and corresponding control by one-way ANOVA for the significant differences. The differences between groups were considered as statistically significant at *p*-value < 0.05.

## 3. Results and Discussion

### 3.1. Identification of the Common Peaks and HPLC Fingerprint Similarity Analysis

**Figure 1:**
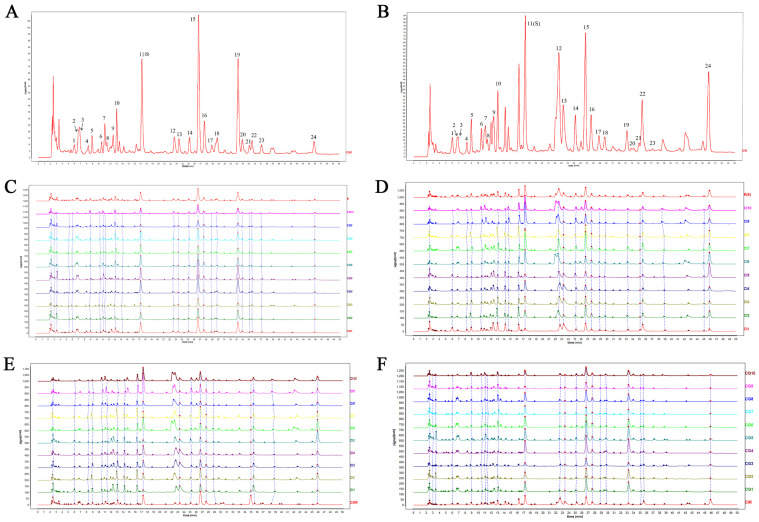
HPLC fingerprints of CG and CI. (**A**) CG reference fingerprint chromatogram (CGR) and 24 common peaks; (**B**) CI reference fingerprint chromatogram (CIR); (**C**) HPLC fingerprints of CG; (**D**) HPLC fingerprints of CI; (**E**) HPLC fingerprints of CI compared to CGR; (**F**) HPLC fingerprints of CG compared to CIR.

HPLC fingerprinting methods for CG and CI were established first. Peaks existing in all chromatograms were assigned as “common peaks”, and 24 common peaks were identified. Peak 11 (s) was chosen as the reference peak to perform method validation (Figure 1A). Precision, reproducibility, and stability were evaluated separately. The RSD of the relative retention time (RRT) and relative peak area (RPA) of each common peak was calculated. The results with relative standard deviation (RSD)s of RRT and RPA are shown in Supporting Materials Tables S2–S7. The RSD of the RRT of each common peak was found to be less than 3.45%, and the RSD of the RPA of each common peak was less than 4.81%. These results suggested that the HPLC fingerprint analysis method was consistent with the requirements.

Ten batches of CG and CI sample chromatograms were processed separately using the median method to generate a reference fingerprint chromatogram (named CGR and CIR; Figure 1A,B), then the similarities between the CGR and CIR and all samples were calculated separately, and the similarity results are shown in Table 1. The similarity of fingerprints is generally used for quality control of plant materials [25]. Similarity between the 10 batches of CG samples and the CGR all were in the range of 0.900–0.991 (Table 1). There was higher similarity within groups, which may be due to the consistency of harvesting period, while similarities between CI samples and CGR are both less than 0.706, except CI7 (0.839). The exception to CI7 may be due to the timing of the harvest and geographical environment because it was collected from Jimisar. In turn, the similarity between CG samples and the CIR is less than 0.72; however, the similarity between CI samples and CIR is greater than 0.789. Among them, CI1 and CI5 are less similar to CIR. This may be due to CI being a perennial wild species. Different growing years for the same species can also make a greater difference, but we did not conduct any further research on the growth years of CI samples. Similarly, a high degree of consistency within the CG group may be due to the fact that they are both annual cultivars. Lower similarity between CG and CI indicates a significant chemical difference between CG and CI. To further identify the differential compounds, a non-targeted metabolomics analysis was carried out.

### 3.2. Non-Targeted LC-QTOF-MS Analysis

**Figure 2:**
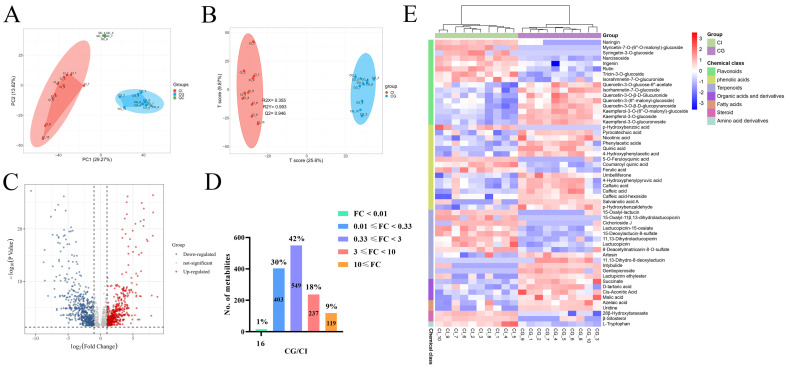
The multivariate analysis of metabolites in CG and CI. (**A**) Principle component analysis (PCA) plot; (**B**) score scatter plot of the OPLS-DA model; (**C**) volcano plot of differential metabolites; (**D**) differential comparison of metabolite intensity; (**E**) heat map of CG and CI. Significantly up- and downregulated metabolites are indicated in red and green, respectively. Those without significant difference between the two groups are indicated in grey. A greater absolute value on the horizontal axis indicates a greater foldchange between CG and CI. A greater value on the vertical axis indicates greater significance.

Plant metabolomics is an emerging technology of systematic science to analysis and identification metabolites of plants with advantages of high throughput, high sensitivity, and wide coverage [26]. Untargeted metabolomics, based on LC-high resolution mass spectrometry (HRMS) combined with multivariate analysis, is able to screen differential compounds in different plants or under various conditions. A metabolome analysis was performed on HPLC-QTOF-MS as the literature mentioned above. Due to the greater response intensity and more peaks generated by negative ion mode, metabolite data of negative models were selected for HPLC-QTOF-MS analysis. The typical base peak chromatograms (BPCs) of CG and CI in the negative ion mode are shown in Figure S1. Based on the macroscopic comparison of metabolic fingerprints, there is a significant difference between CG and CI. Further data analysis with multivariate statistical method for metabolites data of CG and CI were performed. To monitor stability of the LC-MS system, one QC sample was inserted in the running sequence per five test samples. All QC samples were found to tightly cluster together of the PCA score plot (Figure 2A). These results indicated a good reproducibility of the analytical system.

### 3.3. Multivariate Statistical Analysis

After processing the raw data for alignment, deconvolution, data reduction, etc., and further screening by “80% rule” and “30% variation”, 3020 ions were retained. Of these, 1779 were general ions. CG and CI contained 502 and 739 characteristic ions, respectively. The normalized abundance of was used as the input for further chemometrics analysis. Both PCA and OPLS-DA were widely used multivariate analysis methods in metabolomics [27]. To evaluate whether the metabolites profiles could effectively distinguish CG and CI samples, PCA was incipiently selected [28]. As the PCA score scatter plots for all samples included the QC samples shown in Figure 2A, the principal components 1 and 2 explained 29.27% and 13.62% of total variances, respectively, and distinct differences were observed among the CG and CI in the PCA score plot.

OPLS-DA is a supervised model which is used to filter out random noises, distinguish differences, and improve validity and analytical ability of the model. Hence, the OPLS-DA model has better classification efficiency than the PCA model. The score scatter plot inferred from the inter-group comparison of CG and CI samples is shown in Figure 2B. All the samples could be clustered individually in the OPLS-DA score plots. The result was basically consistent with that of the PCA. The CG samples were found to be completely separated from the CI samples, suggesting a significant difference in chemical ingredients. The resulting values of R2Y(cum) and Q2(cum) were 0.993 and 0.946, respectively, which demonstrated that OPLS-DA models were stable and reliable.

VIP was a common method for evaluating the contribution of variables in OPLS-DA. To select the chemical markers, the ions were further screened based using VIP value. As shown in the Figure 2C volcano map, a total of 1324 ions were screened. The higher the VIP value for the ions was, the more far it is away from the origin of volcano plot. This included 419 upregulated (VIP > 1, *p* < 0.05 and FC > 3.0) and 356 downregulated (VIP > 1, *p* < 0.05 and FC < 0.33). About 42% of these metabolites showed no difference between two species (Figure 2D).

### 3.4. Identification of Chemical Markers

The identification of the components in CG and CI extraction was identified by HPLC-Q-TOF-MS based on the exact molecular weight of the parent and fragment ions, and the information for the ions was compared against the published literature and publicly available online metabolite databases Metlin, MassBank, and HMDB [17] to identify the compounds. Based on the criteria for determining the differential metabolites, 59 compounds (chemical markers) were identified as shown in Table 2. There were 20 phenolic acids (**3**, **5**, **8–12**, **14–16**, **20–23**, **25–26**, **28**, **30–31**, **39**), 16 flavonoids (**24**, **36**, **41–54**, **59**), 15 terpenoids (**7**, **17–19**, **27**, **29**, **32–35**, **37–38**, **55**, **57–58**), 4 organic acids and derivatives (**2**, **4**, **6**, **13**), 2 steroids (**40**, **56**), 1 fatty acid (**48**), and 1 amino acid derivative (**1**).

As shown in Figure 2D, heat maps were employed to show the relative intensity of differential chemical markers in CG and CI. The high contents are represented by red squares, while the low ones are represented by green squares. The profile of the chemical markers was able to distinguish CI from CG. Among all compounds, CG possessed higher contents of phenolic acids, organic acids and derivatives, fatty acids, quercetin, and kaempferol and its derivatives. As for CI, the higher contents of terpenoids and steroids were determined. It is worth mentioning that two sesquiterpenoids, 15-deoxylactucin-8-sulfate (**37**) and 8-deacetylmatricarin-8-O-sulfate (**38**), with higher abundances in CI than those in CG, might be used for rapid distinguishing CI from CG by LC-MS. In addition, the much higher levels of kaempferol-3-O-glucuronoside (**46**) and caffeic acid-hexoside (**15**) might be the chemical characters of CG compared to CI.

### 3.5. In Vitro Activity Assay

Based on the difference of chemical composition between CG and CI, the antioxidative and hypoglycemic activities of CG and CI were further compared. The antioxidative activity was determined by DPPH and ABTS assay, while the hypoglycemic activity was assessed by PTG assay. The free radical scavenging ability and hypoglycemic activity of the two samples were concentration-dependent (Figures S2–S4).

As shown in Figure S2, the IC_50_ of the 10 batches of CI samples were all in the range of 105.428 μg/mL−159.831 μg/mL. The IC_50_ of CG samples were in the range of 130.347 μg/mL−252.889 μg/mL. L(+)-ascorbic acid (Vc) (positive control) possessed an IC_50_ value of 5.215 μg/mL. The IC_50_ value of CI is relatively lower than CG, which indicated a higher antioxidative activity compared to CG. The results of ABTS experiment were similar (Figure S3). As for ABTS+ radical scavenging capability, IC_50_ values of CG and CI were in the range from 208.938 μg/mL to 464.131 μg/mL and 136.914 μg/mL to 273.541 μg/mL, respectively. Vc showed an IC_50_ value of 8.649 μg/mL. Taken together, CI extractions showed better antioxidant activity than CG extractions.

**Figure 3:**
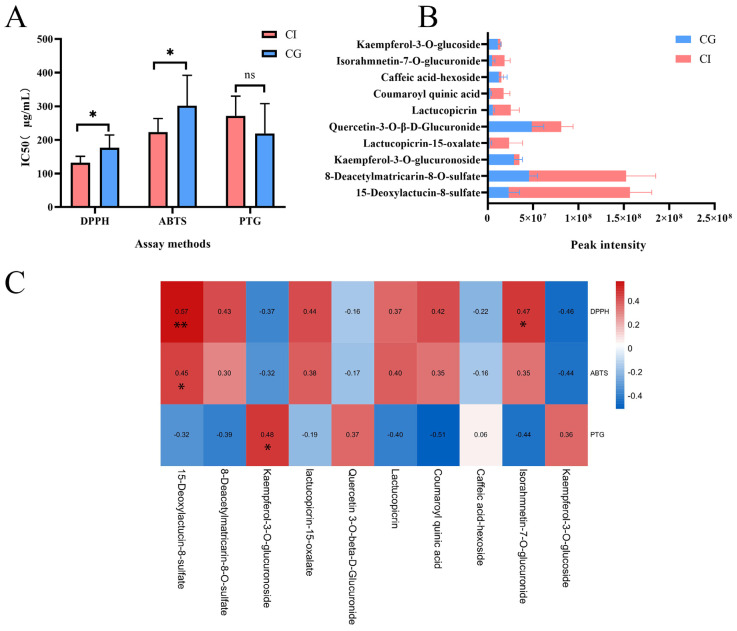
Correlation analysis between activities in vitro and abundance of compounds in CG and CI. (**A**) Comparison of antioxidant activities and hypoglycemic activities between CG and CI evaluated by IC_50_. ns *p* > 0.05, * *p* < 0.05 compared with CI. (**B**) The peak intensity of TOP10 components. (**C**) Spearman’s correlation coefficients to each bioactive compound detected by HPLC. * *p* < 0.05, ** *p* < 0.01 compared with peak intensity.

α-Glycosidase is an important enzyme for digesting carbohydrates. Inhibiting α-glucosidase is believed to be one of the most effective approaches for diabetes care [29]. As shown in Figure S4, CG and CI showed a dose-dependent inhibition activity against α-glucosidase with IC_50_ values of 70.356 μg/mL–352.058 μg/mL of CG and 196.033 μg/mL–364.416 μg/mL of CI. The results suggested that CG extractions had better hypoglycemic activity compared to CI. In order to compare the results more visually, statistical analysis on the antioxidative activity and hypoglycemic activity results of CG and CI were carried out respectively (Figure 3A). IC_50_ values of CI and CG showed significant differences in DPPH and ABTS assays (*p* < 0.05). CG showed better activity in hypoglycemic activity than CI without significant difference.

In order to further explore the material base for antioxidative and hypoglycemic activities, a bivariate correlation analysis was conducted. We excluded trace components, and screened the top ten chemical components with the largest content difference between CG and CI. The main components were sesquiterpenes and flavonoids, as shown in Figure 3B. CI is rich in 15-deoxylactucin-8-sulfate, 8-deacetylmatricarin-8-O-sulfate, lactucopicrin-15-oxalate, lactucopicrin, coumaroyl quinic acid, caffeic acid-hexoside, and isorahmnetin-7-O-glucuronide. Kaempferol-3-O-glucuronoside, quercetin-3-O-β-D-Glucuronide, caffeic acid-hexoside, and kaempferol-3-O-glucoside levels in CG are higher than those in CI.

The bivariate correlation analysis (Figure 3C) presented a significant (*p* < 0.05) positive correlation of 15-deoxylactucin-8-sulfate, 8-deacetylmatricarin-8-O-sulfate, and isorahmnetin-7-O-glucuronide to DPPH and ABTS (r-value from 0.296 to 0.570), which declared that the two compounds contributed to the antioxidative activity of CI. Additionally, the significant (*p* < 0.05) positive correlation of kaempferol-3-O-glucuronoside to PTG (r = 0.483). Therefore, sesquiterpenoids 15-deoxylactucin-8-sulfate, 8-deacetylmatricarin-8-O-sulfate, and flavonoids such as kaempferol-3-O-glucuronoside caused the significant (*p* < 0.05) differences of antioxidant activities and hypoglycemic activities between CG and CI.

### 3.6. Composition Preparation and Pharmacodynamic Evaluation

#### 3.6.1. Composition Preparation Using Preparative-HPLC

Based on the above research, in order to further confirm the correlation between chemical composition and bioactivities, the top three components with the largest content difference between CG and CI were separated and prepared by preparative-HPLC. We separated and enriched 15-deoxylactucin-8-sulfate and 8-deacetylmatricarin-8-O-sulfate in CI and kaempferol-3-O-glucoside (CGA) in CG by preparative liquid chromatography. Due to the low separation degree between 15-deoxylactucin-8-sulfate and 8-deacetylmatricarin-8-O-sulfate, those were selected as a mixture named CIA, and CIA is the strongest correlation to the antioxidative activity tested by DPPH and ABTS assays, and CGA is the strongest correlation to the hypoglycemic activity tested by PTG assay. The purity of CGA was above 95%, and that of CIA was above 80%, as shown in Figure S5. Furthermore, we evaluated the antioxidative and hypoglycemic efficacy of the two samples using larval zebrafish.

#### 3.6.2. Effect of CG and CI on H_2_O_2_-Induced Larval Zebrafish Injury Model In Vivo

**Figure 4:**
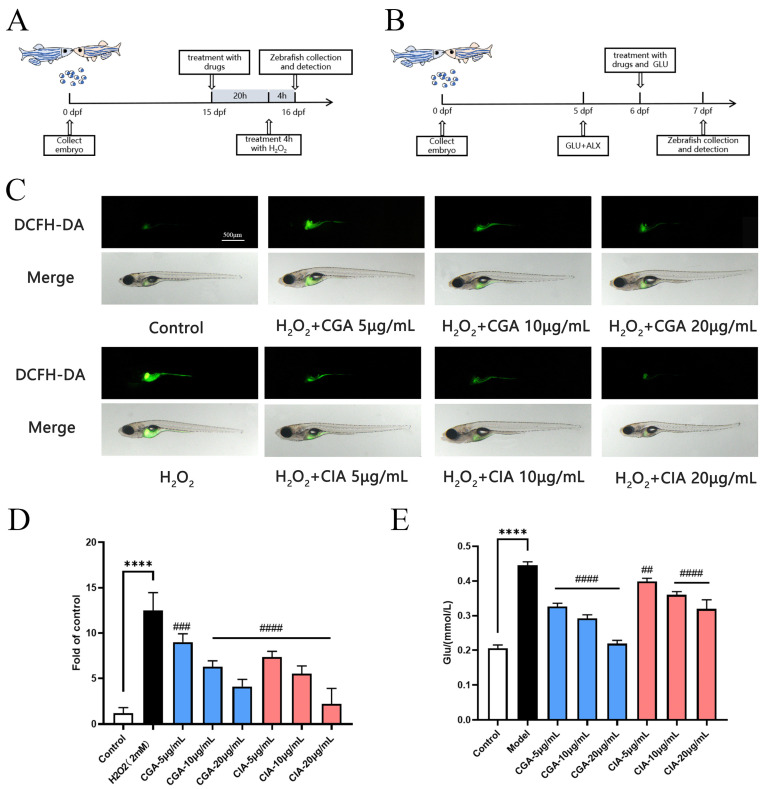
Effect of CGA and CIA on antioxidative and hypoglycemic activities in larval zebrafish. (**A**) Experimental outline of H_2_O_2_ induced larval zebrafish. (**B**) Experimental outline of GLU+ALX induced larval zebrafish. (**C**) ROS production showed in fluorescence image and merged with light field image. (**D**) ROS level quantitated by fluorescence quantitative data. (**E**) Glucose levels of each treated larval group. Bar indicates means ± SD. **** *p* < 0.0001 compared with control; ## *p* < 0.01, ### *p* < 0.001, #### *p* < 0.0001 compared with model. *p* < 0.05 was considered as statistical significance calculated by ANOVA followed by Tukey’s test.

To confirm the antioxidative effect of CGA and CIA, an H_2_O_2_-induced larval zebrafish model was used to assess the activity of their ROS scavenging capability in vivo (Figure 4A). ROS plays an important role in oxidation stress [30,31]. To examine the intracellular ROS levels, DCFH-DA probe, a reactive oxygen-sensitive dye, can be used to detect intracellular ROS generation [32]. As shown in Figure 4C, compared with the control group, fluorescent signals were indeed enhanced in the fishes’ abdomens in the model group, indicating that ROS was formed in the larvae in the presence of free radical generator (H_2_O_2_) and mainly located in the liver and gastrointestinal tissue. This suggests that ROS can cause liver damage. Zebrafish larvae pre-treated with CGA or CIA both showed a lower fluorescent intensity compared to model group. As the corresponding quantified result of ROS shown in Figure 4D, the CGA and CIA (5, 10 and 20 μg/mL) both reduced the liver fluorescent intensity of zebrafish larvae induced by H_2_O_2_ with a dose-dependent pattern. The efficacy of CIA was better than that of CGA at the same dose. The ROS levels of the larvae in high CIA exposure group (20 μg/mL) were close to those in the control group, which demonstrated that CGA and CIA exhibit in vivo free radical scavenging activity. It was consistent with the previous results in DPPH and ABTS assays. CIA plays an important role in antioxidant effect of *Cichorium intybus* L. and is used to protect the liver from damage. The result accorded with the previous report which stated that kaempferol and its derivatives (CGA) have good antioxidant properties in zebrafish larvae [33,34].

#### 3.6.3. Effects of CG and CI on Hypoglycemic Effect Induced by High GLU and ALX in Zebrafish Model

In recent years, zebrafish have shown a significant potential as in vivo models for diabetes-related research. Based on previous study, the combination of GLU and ALX can construct the zebrafish model of insulin-dependent diabetes mellitus. Here, we used 10mg/mL GLU combined with 0.025 μM ALX to induce a diabetes model in zebrafish (Figure 4B). Our results demonstrated that the larvae raised in GLU+ALX solution (Figure 4E) showed increasing of blood glucose levels compared to the control, indicating that the high GLU and ALX caused blood glucose disorder in zebrafish. Treatment with the CGA and CIA resulted in a concentration-dependent decrease in blood glucose of larvae. Compared to CIA, CGA was able to reduce the hyperglycemia of zebrafish more strongly. The results accorded with the previous report which stated that CGA could attenuated blood glucose disorders [35]. The results also implied that CG might be beneficial for diabetics as a dietary supplement. As far as we know, it is the first time to study the hypoglycemic effect of CIA.

## 4. Conclusions

In this study, the HPLC fingerprint of CG and CI is significantly different and can be easily and quickly distinguished. Comparison of metabolic profiles between CG and CI by HPLC-QTOF-MS clarified that CG is rich in phenolic acids, organic acids and derivatives, fatty acids, and flavonoids, while the contents of terpenoids and steroids are higher in CI. Although the types of chemicals they contain are overlapping, there are significant differences in the content of several components. Higher contents of 15-deoxylactucin-8-sulfate and 8-deacetylmatricarin-8-O-sulfate in CI and kaempferol-3-O-glucuronoside and caffeic acid-hexoside in CG might be used for rapid identification of them from each other. In terms of pharmacodynamics comparison, CG possesses better hypoglycemic activity, while CI is better at antioxidant effects. Taken above, in consideration of functional food product development, CG is more suitable for hypoglycemic or obesity-alleviating products, while CI can be used in the development of hepatoprotective products. However, further understanding of CG and CI’s hepatoprotective and hypoglycemic activities is needed in more animal models and even clinical trials for their further application. Additionally, the differential compounds between CG and CI may also contribute to their difference in multiple bioactivities, which also provides a novel interest for investigating the bioactivity of the compounds CG and CI in various aspects in future.

## Figures and Tables

**Table 1:** Results of similarity evaluation of HPLC fingerprint between *Cichorium glandulosum* Boiss et. Huet and *Cichorium intybus* L.

Sample Number	Similarity		Sample Number	Similarity	
	CGR	CIR		CIR	CGR
CG1	0.991	0.642	CI1	0.789	0.521
CG2	0.983	0.600	CI2	0.946	0.706
CG3	0.916	0.741	CI3	0.929	0.646
CG4	0.981	0.600	CI4	0.914	0.588
CG5	0.982	0.614	CI5	0.801	0.445
CG6	0.973	0.674	CI6	0.897	0.557
CG7	0.954	0.723	CI7	0.929	0.839
CG8	0.983	0.679	CI8	0.946	0.706
CG9	0.900	0.626	CI9	0.905	0.689
CG10	0.968	0.708	CI10	0.899	0.626

**Table 2:** Groups of the compounds identified by HPLC-ESI-Q-TOF-MS in CG and CI.

NO.	Compound Name	Elemental Composition	t_R_ (min)	Observed [M-H]^-^	Product Ions (*m/z*)	Chemical Class
1	L-Tryptophan	C_11_H_12_N_2_O_2_	2.36	203.0188	159.03	Amino acid derivatives
2	Malic acid	C_4_H_6_O_5_	2.76	133.0175	71.01, 115.00	Organic acids and derivatives
3	Ferulic acid	C_10_H_10_O_4_	2.79	193.0277	160.84, 117.02	Phenolic acids
4	Cis-Aconitic acid	C_6_H_6_O_6_	2.82	173.0154	85.02, 129.04	Organic acids and derivatives
5	Quinic acid	C_7_H_12_O_6_	2.91	191.0225	111.00, 127.03, 173.04	Phenolic acids
6	Succinate	C_4_H_6_O_4_	3.59	117.0159	59.01, 99.02	Organic acids and derivatives
7	Gentiopicroside	C_16_H_20_O_9_	3.77	355.1281	191.01, 177.07	Terpenoids
8	Umbelliferone	C_9_H_6_O_3_	3.82	161.0450	117.05	Phenolic acids
9	Salvianolic acid A	C_26_H_22_O_10_	5.36	493.1242	295.06	Phenolic acids
10	Caftaric acid	C_13_H_12_O_9_	6.91	311.0416	135.04, 179.03, 87.00	Phenolic acids
11	4-Hydroxyphenylpyruvic acid	C_9_H_8_O_4_	6.92	179.0340	135.04	Phenolic acids
12	Phenylacetic acid	C_8_H_8_O_2_	6.99	135.0441	91.05	Phenolic acids
13	D-tartaric acid	C_4_H_6_O_6_	7.07	149.0086	87.00, 103.01	Organic acids and derivatives
14	Pyrocatechuic acid	C_7_H_6_O_4_	7.16	153.0226	109.02	Phenolic acids
15	Caffeic acid-hexoside	C_15_H_18_O_9_	8.65	341.0885	179.03, 135.04	Phenolic acids
16	Cichoriin	C_15_H_16_O_9_	8.98	339.0759	177.01, 133.02	Phenolic acids
17	Intybulide	C_15_H_16_O_5_	9.19	275.0936	194.90, 150.90	Terpenoids
18	Lactupicrin ethylester	C_25_H_28_O_8_	9.48	455.1557	275.08	Terpenoids
19	8-Omrthylsenecioylaustricin	C_21_H_26_O_5_	10.57	357.0709	194.9, 173.0, 217.0	Terpenoids
20	Chlorogenic acid	C_16_H_18_O_9_	11.03	353.0935	309.09	Phenolic acids
21	4-Hydroxyphenylacetic acid	C_8_H_8_O_3_	12.01	151.0397	151.04, 107.05	Phenolic acids
22	Esculetin	C_9_H_6_O_4_	12.99	177.0221	133.02,131.01	Phenolic acids
23	Caffeic acid	C_9_H_8_O_4_	13.31	179.0337	135.04	Phenolic acids
24	Irigenin	C_18_H_16_O_8_	13.71	359.0815	96.96	Flavonoids
25	p-Hydroxybenzaldehyde	C_7_H_6_O_2_	13.85	121.0294	93.03	Phenolic acids
26	Nicotinic acid	C_6_H_5_NO_2_	13.87	122.0316	94.05	Phenolic acids
27	Cichorioside J	C_22_H_28_O_10_	14.48	451.0832	59.01, 423.07, 361.01	Terpenoids
28	Coumaroyl quinic acid	C_16_H_18_O_8_	14.50	337.1127	191.05	Phenolic acids
29	15-Oxalyl-lactucin	C_17_H_16_O_8_	14.92	347.0851	213.09, 257.08, 275.09	Terpenoids
30	Caffeoylquinic acid	C_16_H_18_O_9_	16.33	353.0341	191.0, 179.0	Phenolic acids
31	Cichoric acid	C_22_H_18_O_12_	17.37	473.0713	179.03	Phenolic acids
32	11,13-Dihydro-8-deoxylactucin	C_15_H_18_O_4_	19.94	261.1068	229.08	Terpenoids
33	Artesin	C_15_H_22_O_5_	20.71	281.1305	201.00	Terpenoids
34	11β,13-dihydrolactucin	C_15_H_18_O_5_	21.31	277.0333	260.18	Terpenoids
35	15-Oxalyl-11β,13-dihydrolactucopicrin	C_25_H_24_O_11_	21.94	483.1583	325.05, 179.03	Terpenoids
36	Myricetin-7-O-(6″-O-malonyl)-glucoside	C_24_H_22_O_16_	22.00	565.1735	521.1	Flavonoids
37	15-Deoxylactucin-8-sulfate	C_15_H_16_O_7_S	22.81	339.0589	96.96, 79.95	Terpenoids
38	8-Deacetylmatricarin-8-O-sulfate	C_15_H_18_O_7_S	23.39	341.0715	96.95, 79.95	Terpenoids
39	Chlorogenic acid B	C_25_H_24_O_12_	24.99	515.1215	191.05	Phenolic acids
40	28β-Hydroxytaraxaste	C_30_H_52_O	26.14	441.0878	277.03, 295.04, 259.03	Steroid
41	Quercetin-3-O-β-D-Glucuronide	C_21_H_18_O_13_	26.53	477.0717	301.03	Flavonoids
42	Quercetin-3-O-β-D-glucopyranoside	C_21_H_20_O_12_	27.42	463.0915	300.02	Flavonoids
43	Rutin	C_27_H_30_O_16_	27.52	609.1516	300.02	Flavonoids
44	Quercetin 3-O-(6″-malonyl-glucoside)	C_24_H_22_O_15_	29.61	549.0928	505.09, 300.02	Flavonoids
45	Quercetin 3-O-(6″-acetyl-glucoside)	C_23_H_22_O_13_	29.79	505.1033	301.03, 59.01	Flavonoids
46	Kaempferol-3-O-glucuronoside	C_21_H_18_O_12_	33.08	461.0787	285.04	Flavonoids
47	Kaempferol-3-O-glucoside	C_21_H_20_O_11_	33.74	447.0956	284.03, 285.00	Flavonoids
48	Azelaic acid	C_9_H_16_O_4_	34.46	187.0969	125.09	Fatty acids
49	Syringetin-3-O-glucoside	C_23_H_24_O_13_	34.72	507.1187	151.00, 303.05	Flavonoids
50	Isorhamnetin-7-O-glucoside	C_22_H_23_O_12_	34.94	477.1070	314.04	Flavonoids
51	Isorahmnetin-7-O-glucuronide	C_22_H_20_O_13_	35.25	491.0934	315.04	Flavonoids
52	Narcissoside	C_28_H_32_O_16_	35.60	623.1667	315.04, 314.04, 300.02	Flavonoids
53	Naringin	C_27_H_32_O_14_	35.81	625.1574	271.10, 151.00	Flavonoids
54	Kaempferol-3-O-(6″-O-malonyl)-glucoside	C_24_H_22_O_14_	37.00	533.0981	285.03, 489.09	Flavonoids
55	Lactucopicrin-15-oxalate	C_25_H_22_O_10_	42.96	481.1142	213.09, 257.08, 151.03	Terpenoids
56	β-Sitosterol	C_29_H_50_O	45.03	413.1624	395.07	Steroid
57	11,13-Dihydrolactucopicrin	C_23_H_24_O_7_	45.12	411.1515	215.10, 151.04	Terpenoids
58	Lactucopicrin	C_23_H_22_O_7_	45.82	409.1385	213.09, 257.08, 275.09	Terpenoids
59	Tricin-3-O-glucoside	C_23_H_24_O_12_	45.85	491.1452	329.23, 311.21	Flavonoids

## Data Availability

The data presented in this study are available upon request from the corresponding authors.

## Supplementary Materials

The following supporting information can be downloaded at: https://www.mdpi.com/article/10.3390/foods12040901/s1, Table S1: Sources of materials tested; Table S2: Relative retention time of common peaks in replicate experiments; Table S3: Relative peak area of common peaks in replicate experiments; Table S4: Precision experiment common peak relative retention time; Table S5: Precision experiment common peak relative peak area; Table S6: Relative retention time of common peaks in stability experiments; Table S7: Stability experiment common peak relative peak area; Figure S1: The base peak chromatograms (BPCs) in negative mode CI (A) and CG (B); Figure S2: Scavenging abilities (%) of CG and CI at different concentrations, determined by DPPH assay. L(+)-ascorbic acid (Vc); Figure S3: Scavenging abilities (%) of CG and CI at different concentrations, determined by DPPH assay. L(+)-ascorbic acid (Vc); Figure S4: Inhibitory effects of CG and CI on α-glucosidase activities; Figure S5: Purity determination of the components obtained from preparative HPLC. (A) CGA peak with >95% purity (UV 258 nm); (B) HPLC analysis of CG after removal of CGA; (C) HPLC analysis of CG; (D) CIA peak with >80% purity (UV 258 nm); (E) HPLC analysis of CI after removal of CIA; F: HPLC analysis of CI.
