# Supplementary material for: Chemometric Discrimination of Cichorium glandulosum Boiss. et Huet and Cichorium intybus L. via Their Metabolic Profiling, Antioxidative, and Hypoglycemic Activities
Source: Foods. 2023 Feb 20;12(4):901. doi: 10.3390/foods12040901 (PMC9957518; doi:10.3390/foods12040901)
Supplement: Supplementary file 1 [file foods-12-00901-s001.zip › foods-2196944-supplementary.pdf]

Supplementary Material

Supplementary Table S1. Sources of materials tested.

Supplementary Table S2. Relative retention time of common peaks in replicate experiments.

Supplementary Table S3. Relative peak area of common peaks in replicate experiments.

Supplementary Table S4. Precision experiment common peak relative retention time.

Supplementary Table S5. Precision experiment common peak relative peak area.

Supplementary Table S6. Relative retention time of common peaks in stability experiments.

Supplementary Table S7. Stability experiment common peak relative peak area.

Supplementary Figure S1. The base peak chromatograms (BPCs) in negative mode CI (A) and CG (B).

Supplementary Figure S2. Scavenging abilities (%) of CG and CI at different concentrations, determined by DPPH assay. L(+)-ascorbic acid (Vc).

Supplementary Figure S3. Scavenging abilities (%) of CG and CI at different concentrations, determined by DPPH assay. L(+)-ascorbic acid (Vc).

Supplementary Figure S4. Inhibitory effects of CG and CI on α-glucosidase activities.

Supplementary Figure S5. Purity determination of the components obtained from preparative HPLC. (**A**) CGA peak with ＞95% purity (UV 258 nm) ; (**B**) HPLC analysis of CG after removal of CGA; (**C**) HPLC analysis of CG; (**D**) CIA peak with ＞80% purity (UV 258 nm) ; (**E**) HPLC analysis of CI after removal of CIA; F: HPLC analysis of CI.

Supplementary Table S1. Sources of materials tested.

NO.	Name	Collection	Soure
CG1	*Cichorium glandulosum* Boiss. et Huet	2015.07	Jimisar County
CG2	*Cichorium glandulosum* Boiss. et Huet	2015.07	Jimisar County
CG3	*Cichorium glandulosum* Boiss. et Huet	2017.07	Jimisar County
CG4	*Cichorium glandulosum* Boiss. et Huet	2017.06	Jimisar County
CG5	*Cichorium glandulosum* Boiss. et Huet	2017.06	Jimisar County
CG6	*Cichorium glandulosum* Boiss. et Huet	2017.06	Jimisar County
CG7	*Cichorium glandulosum* Boiss. et Huet	2017.07	Manasi County
CG8	*Cichorium glandulosum* Boiss. et Huet	2017.06	Jimisar County
CG9	*Cichorium glandulosum* Boiss. et Huet	2017.08	Urumqi city
CG10	*Cichorium glandulosum* Boiss. et Huet	2017.09	Purchased in CFDR
CI1	*Cichorium intybus* L.	2020.06	Urumqi city
CI2	*Cichorium intybus* L.	2020.06	Urumqi city
CI3	*Cichorium intybus* L.	2020.06	Urumqi city
CI4	*Cichorium intybus* L.	2020.06	Urumqi city
CI5	*Cichorium intybus* L.	2020.06	Jimisar County
CI6	*Cichorium intybus* L.	2020.06	Urumqi city
CI7	*Cichorium intybus* L.	2017.10	Jimisar County
CI8	*Cichorium intybus* L.	2018.09	Urumqi city
CI9	*Cichorium intybus* L.	2020.09	Urumqi city
CI10	*Cichorium intybus* L.	2020.09	Urumqi city

Supplementary Table S2. Relative retention time of common peaks in replicate experiments.

NO.	1	2	3	4	5	6	RSD （%）
1	0.351	0.347	0.355	0.347	0.342	0.348	1.27
2	0.389	0.387	0.392	0.388	0.383	0.388	0.74
3	0.401	0.398	0.404	0.399	0.395	0.399	0.72
4	0.477	0.477	0.483	0.479	0.474	0.480	0.60
5	0.521	0.517	0.524	0.520	0.514	0.521	0.67
6	0.624	0.616	0.623	0.618	0.612	0.620	0.70
7	0.641	0.639	0.646	0.642	0.638	0.645	0.51
8	0.662	0.660	0.665	0.663	0.658	0.666	0.44
9	0.731	0.724	0.728	0.726	0.722	0.730	0.48
10	0.754	0.754	0.757	0.757	0.754	0.760	0.34
11(S)	1.000	1.000	1.000	1.000	1.000	1.000	0.00
12	1.307	1.309	1.307	1.310	1.303	1.305	0.20
13	1.373	1.373	1.370	1.374	1.363	1.366	0.33
14	1.439	1.448	1.446	1.453	1.441	1.446	0.36
15	1.531	1.542	1.538	1.549	1.534	1.539	0.41
16	1.580	1.594	1.590	1.604	1.587	1.594	0.49
17	1.657	1.672	1.665	1.681	1.663	1.669	0.50
18	1.705	1.719	1.712	1.730	1.713	1.719	0.49
19	1.896	1.918	1.906	1.933	1.915	1.922	0.67
20	1.932	1.956	1.943	1.972	1.954	1.962	0.72
21	1.997	2.023	2.010	2.042	2.023	2.031	0.79
22	2.024	2.051	2.037	2.070	2.049	2.058	0.79
23	2.114	2.144	2.127	2.165	2.141	2.151	0.84
24	2.595	2.649	2.613	2.673	2.638	2.655	1.09

Supplementary Table S3. Relative peak area of common peaks in replicate experiments.

NO.	1	2	3	4	5	6	RSD （%）
1	0.133	0.135	0.134	0.133	0.136	0.135	0.95
2	0.237	0.250	0.242	0.254	0.253	0.259	3.21
3	0.313	0.320	0.311	0.311	0.309	0.317	1.30
4	0.114	0.113	0.115	0.112	0.114	0.117	1.25
5	0.105	0.103	0.106	0.105	0.106	0.106	1.14
6	0.046	0.048	0.046	0.047	0.049	0.050	3.31
7	0.209	0.214	0.210	0.216	0.214	0.223	2.33
8	0.087	0.095	0.096	0.096	0.097	0.099	4.11
9	0.227	0.220	0.219	0.214	0.205	0.208	3.88
10	0.293	0.298	0.305	0.294	0.285	0.288	2.47
11(S)	1.000	1.000	1.000	1.000	1.000	1.000	0.00
12	0.134	0.132	0.134	0.134	0.131	0.134	1.14
13	0.121	0.119	0.120	0.124	0.124	0.129	2.84
14	0.200	0.206	0.208	0.205	0.205	0.208	1.51
15	1.730	1.777	1.764	1.765	1.774	1.813	1.51
16	0.279	0.289	0.286	0.285	0.289	0.294	1.78
17	0.107	0.111	0.114	0.112	0.111	0.114	2.47
18	0.299	0.326	0.322	0.319	0.320	0.325	3.09
19	1.133	1.156	1.158	1.160	1.163	1.183	1.38
20	0.210	0.214	0.217	0.217	0.218	0.220	1.60
21	0.067	0.069	0.068	0.071	0.073	0.076	4.81
22	0.149	0.156	0.151	0.150	0.150	0.152	1.83
23	0.113	0.116	0.115	0.116	0.113	0.114	1.08
24	0.099	0.105	0.099	0.100	0.102	0.107	3.34

Supplementary Table S4. Precision experiment common peak relative retention time.

NO.	1	2	3	4	5	6	RSD（%）
1	0.348	0.350	0.346	0.319	0.348	0.347	3.45
2	0.389	0.390	0.386	0.386	0.387	0.387	0.38
3	0.400	0.402	0.397	0.398	0.399	0.399	0.39
4	0.482	0.483	0.479	0.478	0.481	0.482	0.41
5	0.523	0.522	0.517	0.516	0.520	0.522	0.52
6	0.618	0.616	0.611	0.612	0.617	0.615	0.44
7	0.644	0.643	0.639	0.640	0.644	0.644	0.35
8	0.664	0.663	0.660	0.659	0.664	0.664	0.32
9	0.725	0.724	0.720	0.721	0.723	0.724	0.25
10	0.757	0.758	0.755	0.754	0.758	0.759	0.25
11(S)	1.000	1.000	1.000	1.000	1.000	1.000	0.00
12	1.315	1.307	1.315	1.301	1.306	1.312	0.45
13	1.370	1.362	1.369	1.352	1.360	1.367	0.51
14	1.457	1.450	1.460	1.436	1.453	1.460	0.61
15	1.550	1.542	1.551	1.523	1.544	1.551	0.69
16	1.606	1.598	1.609	1.576	1.602	1.608	0.77
17	1.683	1.672	1.687	1.645	1.679	1.683	0.91
18	1.733	1.722	1.739	1.693	1.732	1.733	0.96
19	1.932	1.929	1.944	1.887	1.942	1.932	1.09
20	1.972	1.973	1.987	1.926	1.985	1.973	1.12
21	2.041	2.043	2.060	1.993	2.056	2.043	1.17
22	2.066	2.069	2.087	2.018	2.080	2.068	1.18
23	2.158	2.161	2.181	2.106	2.172	2.159	1.21
24	2.661	2.661	2.702	2.594	2.679	2.669	1.35

Supplementary Table S5. Precision experiment common peak relative peak area.

NO.	1	2	3	4	5	6	RSD （%）
1	0.136	0.136	0.137	0.127	0.129	0.131	3.06
2	0.254	0.251	0.252	0.240	0.255	0.251	2.08
3	0.325	0.322	0.318	0.314	0.316	0.313	1.43
4	0.119	0.116	0.116	0.114	0.116	0.113	1.65
5	0.109	0.108	0.108	0.107	0.107	0.107	0.86
6	0.051	0.051	0.049	0.049	0.051	0.049	2.15
7	0.223	0.224	0.223	0.220	0.229	0.224	1.36
8	0.098	0.098	0.095	0.094	0.096	0.094	1.89
9	0.203	0.213	0.217	0.214	0.220	0.202	3.48
10	0.287	0.289	0.291	0.286	0.290	0.291	0.71
11(S)	1.000	1.000	1.000	1.000	1.000	1.000	0.00
12	0.190	0.197	0.180	0.197	0.204	0.184	4.69
13	0.133	0.132	0.129	0.132	0.140	0.132	2.65
14	0.194	0.195	0.195	0.188	0.194	0.189	1.73
15	1.818	1.800	1.801	1.749	1.809	1.778	1.38
16	0.328	0.328	0.330	0.320	0.328	0.323	1.18
17	0.096	0.097	0.096	0.092	0.093	0.105	4.65
18	0.283	0.281	0.281	0.271	0.278	0.288	2.07
19	1.196	1.184	1.194	1.153	1.187	1.176	1.34
20	0.223	0.220	0.224	0.214	0.220	0.221	1.56
21	0.078	0.079	0.079	0.075	0.081	0.079	2.50
22	0.151	0.148	0.150	0.143	0.149	0.148	1.90
23	0.108	0.107	0.108	0.102	0.104	0.104	2.53
24	0.124	0.121	0.123	0.119	0.121	0.119	1.81

Supplementary Table S6. Relative retention time of common peaks in stability experiments.

NO.	1	2	3	4	5	6	RSD (%)
1	0.351	0.347	0.344	0.348	0.348	0.352	0.73
2	0.389	0.387	0.385	0.388	0.388	0.389	0.40
3	0.401	0.399	0.397	0.400	0.400	0.401	0.38
4	0.477	0.478	0.476	0.480	0.480	0.481	0.34
5	0.521	0.518	0.518	0.520	0.520	0.521	0.25
6	0.624	0.617	0.617	0.619	0.616	0.616	0.49
7	0.641	0.641	0.641	0.644	0.642	0.642	0.21
8	0.662	0.662	0.661	0.664	0.662	0.661	0.18
9	0.731	0.726	0.724	0.727	0.725	0.723	0.37
10	0.754	0.756	0.754	0.758	0.757	0.755	0.22
11(S)	1.000	1.000	1.000	1.000	1.000	1.000	0.00
12	1.307	1.307	1.306	1.311	1.311	1.312	0.19
13	1.373	1.371	1.367	1.372	1.371	1.369	0.15
14	1.439	1.446	1.442	1.451	1.453	1.456	0.42
15	1.531	1.541	1.534	1.545	1.549	1.548	0.49
16	1.580	1.595	1.586	1.598	1.604	1.603	0.60
17	1.657	1.672	1.661	1.674	1.680	1.678	0.57
18	1.705	1.721	1.709	1.722	1.727	1.724	0.55
19	1.896	1.920	1.905	1.923	1.926	1.921	0.68
20	1.932	1.959	1.944	1.961	1.966	1.959	0.73
21	1.997	2.028	2.011	2.030	2.034	2.027	0.77
22	2.024	2.056	2.039	2.057	2.061	2.052	0.75
23	2.114	2.148	2.130	2.148	2.152	2.145	0.76
24	2.595	2.644	2.620	2.643	2.649	2.655	0.87

Supplementary Table S7. Stability experiment common peak relative peak area.

NO.	1	2	3	4	5	6	RSD （%）
1	0.133	0.135	0.134	0.132	0.135	0.131	0.98
2	0.237	0.253	0.255	0.251	0.256	0.250	3.03
3	0.313	0.316	0.315	0.316	0.312	0.314	0.55
4	0.114	0.115	0.114	0.116	0.114	0.117	0.91
5	0.105	0.107	0.107	0.106	0.106	0.108	0.78
6	0.043	0.047	0.047	0.047	0.048	0.049	4.40
7	0.209	0.216	0.216	0.216	0.219	0.222	1.62
8	0.087	0.097	0.096	0.097	0.097	0.096	4.41
9	0.227	0.220	0.214	0.204	0.205	0.203	4.64
10	0.293	0.294	0.286	0.273	0.277	0.285	3.32
11(S)	1.000	1.000	1.000	1.000	1.000	1.000	0.00
12	0.134	0.140	0.134	0.129	0.129	0.131	3.49
13	0.121	0.115	0.119	0.123	0.127	0.131	3.60
14	0.200	0.207	0.206	0.205	0.202	0.200	1.58
15	1.730	1.773	1.765	1.767	1.764	1.801	0.98
16	0.279	0.288	0.287	0.286	0.285	0.287	1.29
17	0.107	0.102	0.102	0.109	0.108	0.113	3.13
18	0.299	0.297	0.306	0.302	0.302	0.309	1.09
19	1.133	1.162	1.160	1.159	1.157	1.187	1.05
20	0.210	0.214	0.214	0.215	0.214	0.219	0.89
21	0.067	0.069	0.068	0.071	0.074	0.077	3.96
22	0.149	0.155	0.151	0.150	0.149	0.153	1.76
23	0.113	0.116	0.114	0.114	0.113	0.111	1.25
24	0.099	0.101	0.098	0.103	0.105	0.116	2.80

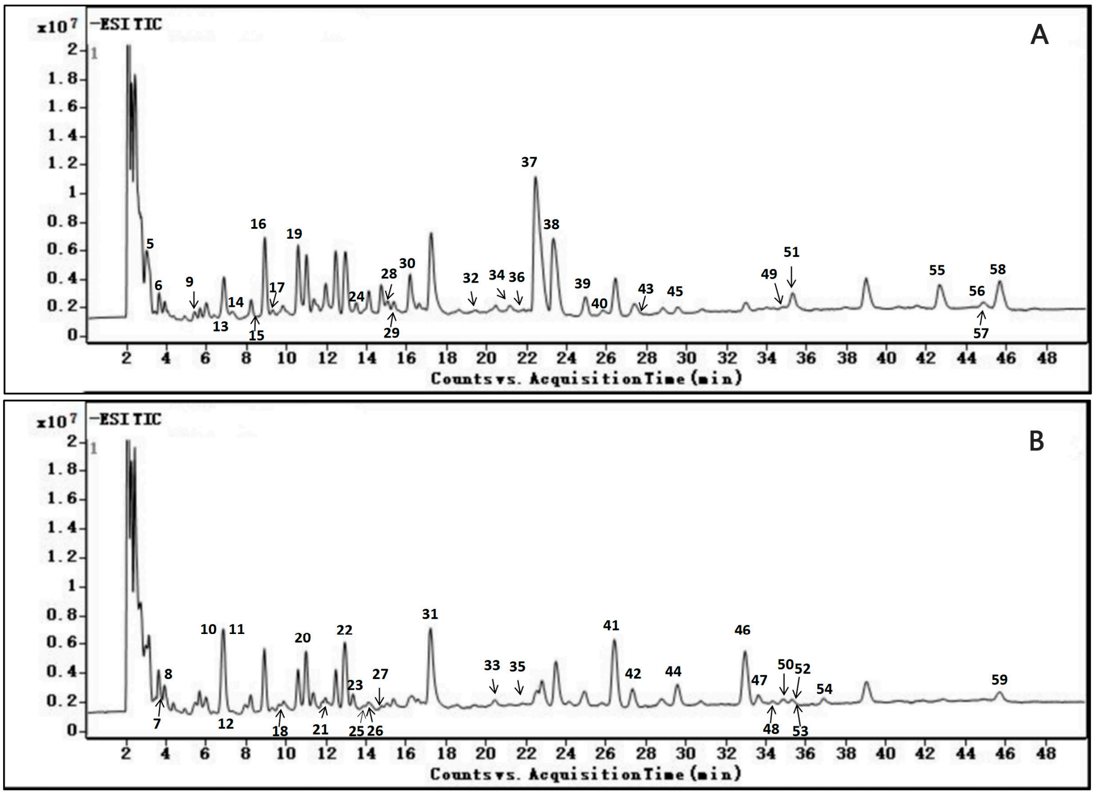

Supplementary Figure S1. The base peak chromatograms (BPCs) in negative mode CI (A) and CG (B).

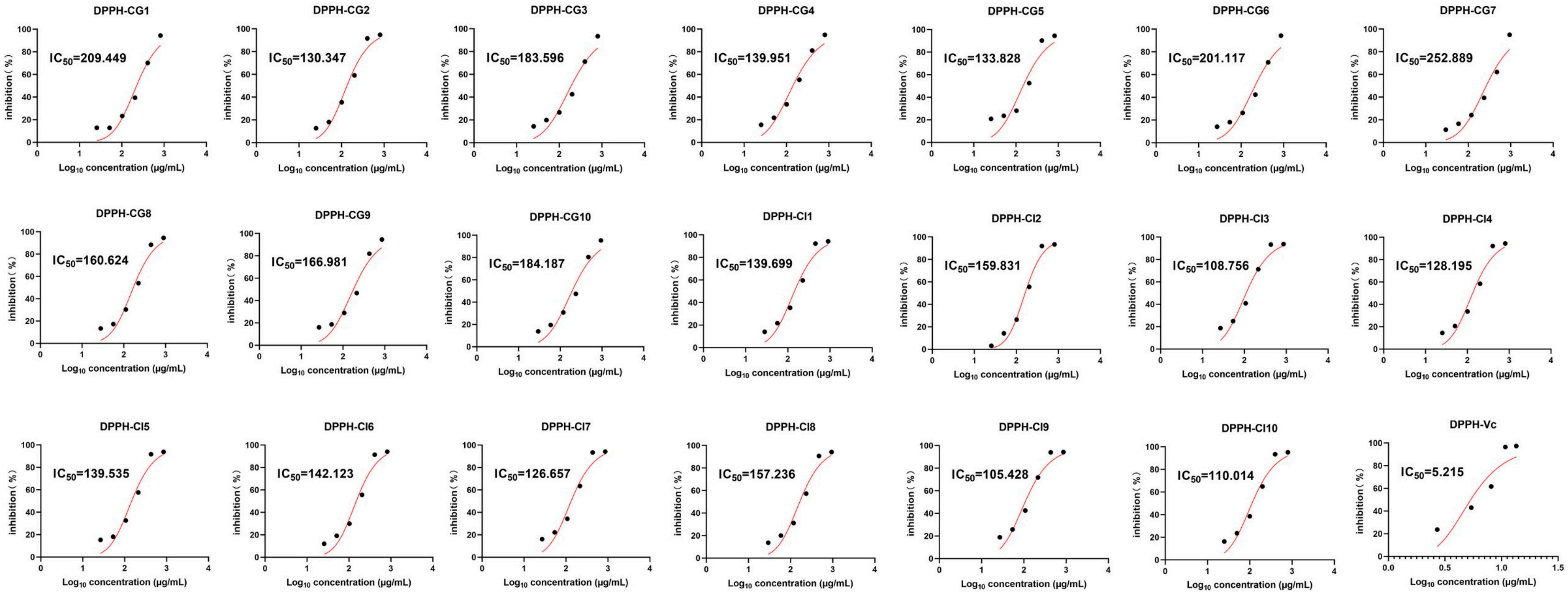

Supplementary Figure S2. Scavenging abilities (%) of CG and CI at different concentrations, determined by DPPH assay. L(+)-ascorbic acid (Vc).

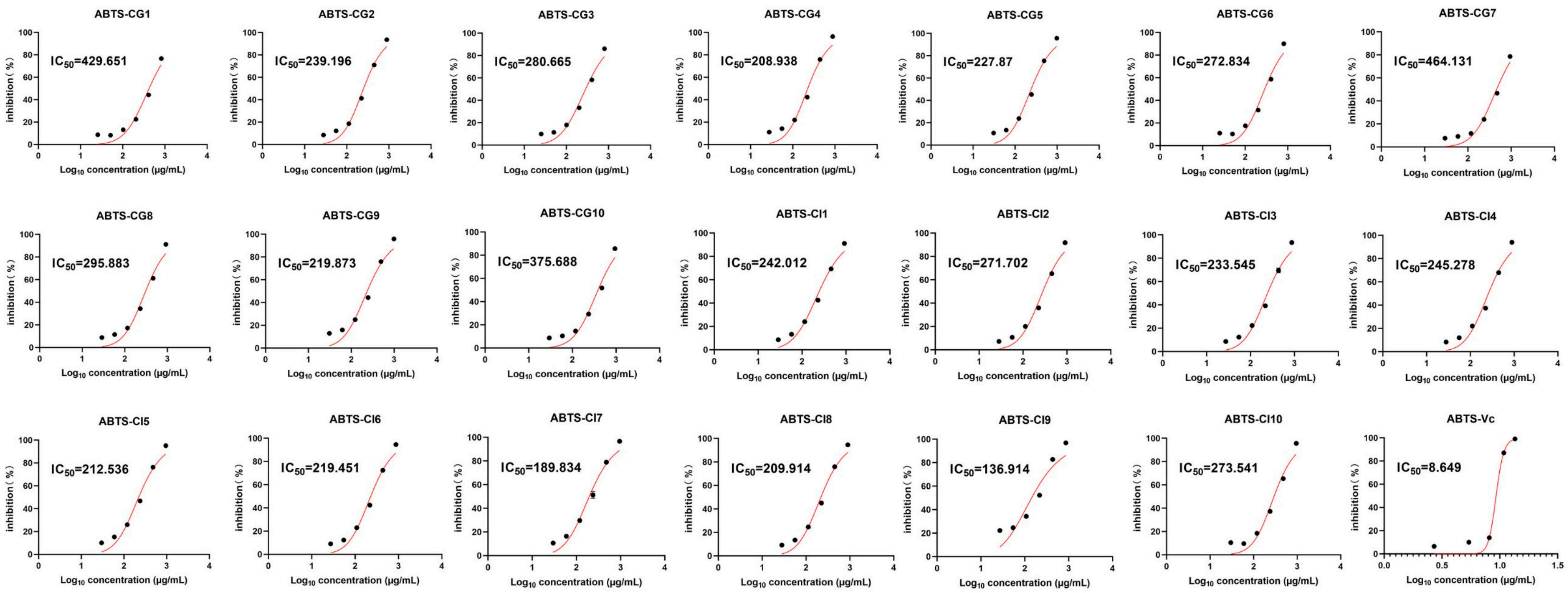

Supplementary Figure S3. Scavenging abilities (%) of CG and CI at different concentrations, determined by ABTS assay. L(+)-ascorbic acid (Vc).

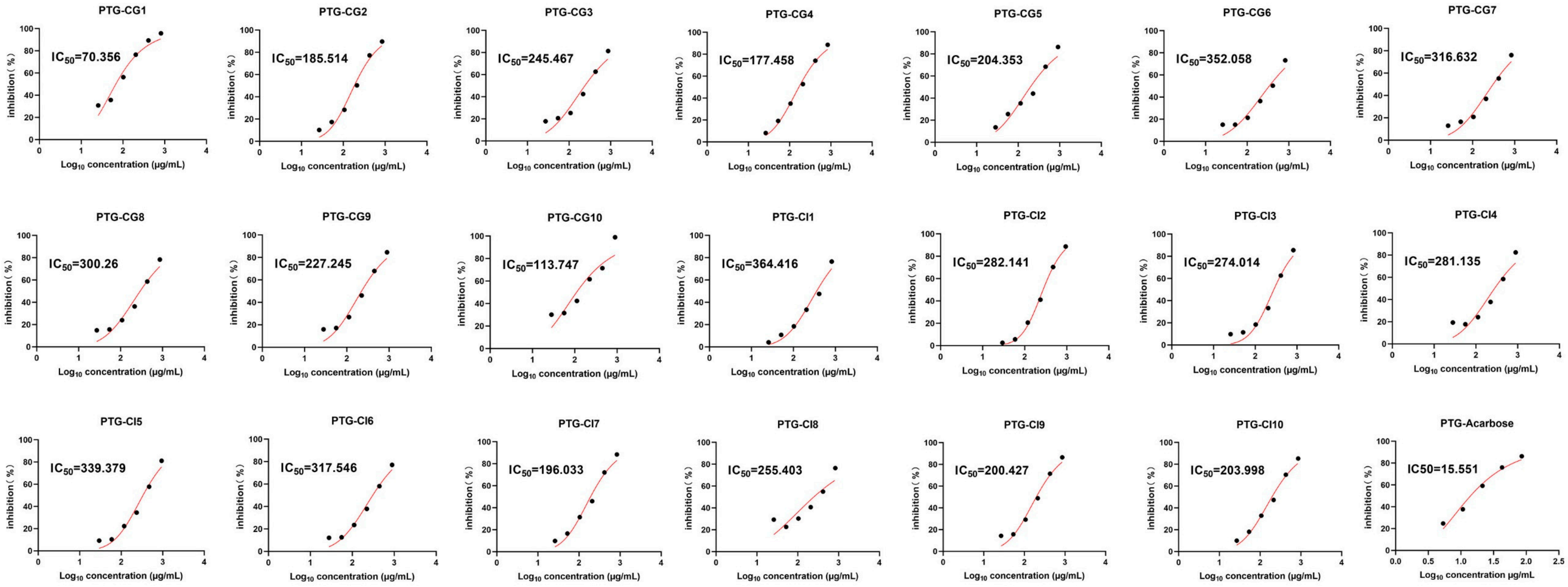

Supplementary Figure S4. Inhibitory effects of CG and CI on α-glucosidase activities.

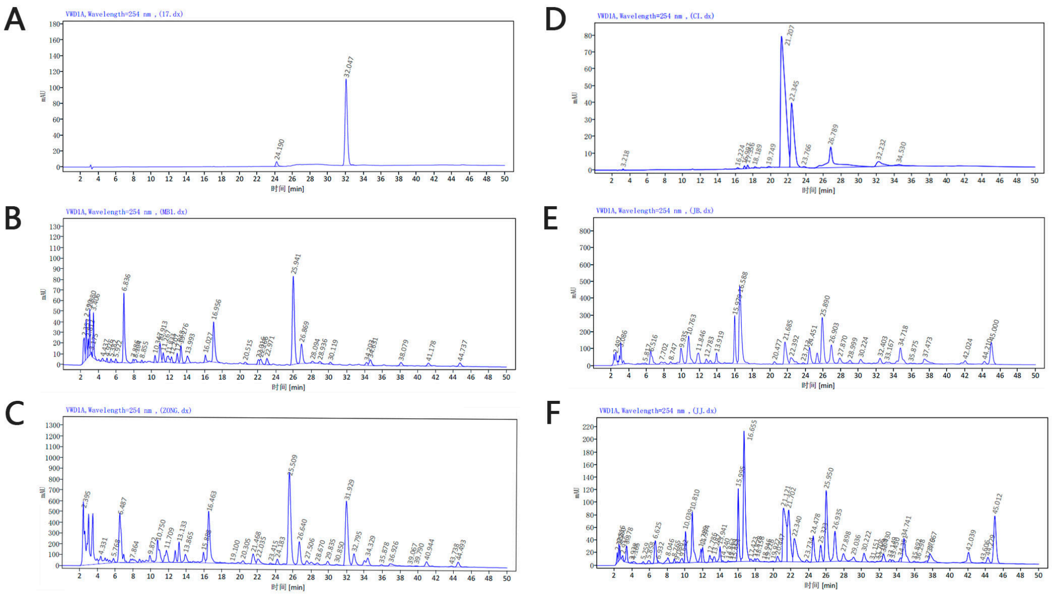

Supplementary Figure S5. Purity determination of the components obtained from preparative HPLC. (**A**) CGA peak with ＞95% purity (UV 258 nm) ; (**B**) HPLC analysis of CG after removal of CGA; (**C**) HPLC analysis of CG; (**D**) CIA peak with ＞80% purity (UV 258 nm) ; (**E**) HPLC analysis of CI after removal of CIA; F: HPLC analysis of CI.
